# Leukocyte *CH25H* is a potential diagnostic and prognostic marker for lung adenocarcinoma

**DOI:** 10.1038/s41598-022-24183-9

**Published:** 2022-12-23

**Authors:** Jun Zhang, Lidong Xu, Jun Gao, Jieyi Li, Xiaokai Zhao, Pengmin Yang, Yujun Ge, Dawei Guo, Zhonghua Liu, Xiyong Wang, Wenchao Gu, Pengli Wang, Xun Hu, Ziying Gong, Daoyun Zhang

**Affiliations:** 1 Department of Cardiothoracic Surgery, The Second Affiliated Hospital of Jiaxing University, 1518 North Huancheng Road, Jiaxing, 314000 China; 2 Jiaxing Key Laboratory of Precision Medicine and Companion Diagnostics, Jiaxing Yunying Medical Inspection Co., Ltd., 153 Huixin Road, Jiaxing, 314000 China

**Keywords:** Non-small-cell lung cancer, Non-small-cell lung cancer

## Abstract

Metastasis, a major challenge during the treatment of lung cancer, causes deterioration in patient health outcomes. Thus, to address this problem, this study aimed to explore the role and contribution of Cholesterol 25-Hydroxylase (*CH25H*) as a potential diagnostic and prognostic marker in lung cancer. Online public databases were used to analyze the expression level, prognostic value, gene-pathway enrichment, and immune infiltration of *CH25H* in lung cancer patients. The Real-Time Quantitative Reverse Transcription PCR (qRT-PCR) was used to analyze and detect the *CH25H* expression levels in leukocytes from lung cancer patients. The expression level of *CH25H* was significantly reduced in lung adenocarcinoma (LUAD), which is associated with a higher disease stage, but not in lung squamous cell carcinoma (LUSC). Kaplan–Meier survival analysis indicated that LUAD patients with low *CH25H* expression had a worse prognosis. Mechanistically, our results showed that in LUAD, *CH25H* may be a regulatory factor affecting the immune cell infiltration level, and the resultant tumor development. Experimental data showed that low expression of *CH25H* in leukocytes was significantly associated with LUAD metastasis (P < 0.01). Our study suggests that *CH25H* may function as a prognostic and risk stratification biomarker for LUAD.

## Introduction

Lung cancer (LC) is the most common malignancy, causing more than 21% of the cancer-related deaths in China^1^. The high recurrence and metastasis rates of LC have worsened the prognosis in patients^2^. Therefore, the discrimination and intervention of treatment techniques in patients with a high risk of metastasis could greatly contribute to the improvements in clinical efficacy and ultimate survival.

Novel approaches, such as liquid biopsy, employing the detection of tumor-derived circulating cell-free DNA/RNA, are powerful clinical strategies in the supervision of cancer progression because of their high sensitivity or specificity^3–5^. However, owing to technical difficulties and costs, these detection approaches are difficult to implement. Therefore, there is a need to develop cheaper and simpler methods and techniques that employ the detection of new biomarkers for predicting LC prognosis. Many small molecules, such as *LKB1*, *RASSF1A*, and *HOXB13,* have been reported to be associated with LC recurrence and metastasis^6–8^. Notably, a recent study showed that low mRNA expression of *CH25H* in leukocytes of melanoma patients is associated with a poor prognosis^9^. The proportion of patients with melanoma having high *CH25H* expression was 54.9% in the non-metastatic group. This was significantly higher than that in the metastatic group (14.8 %). In addition, researchers found that overall survival was significantly higher in patients with high *CH25H* expression than in those with low *CH25H* expression, suggesting that *CH25H* expression may act as an independent prognostic factor for predicting distant metastasis of breast cancer^10^.

However, the expression, prognostic value, and mechanism of *CH25H* in lung cancer have not been reported. This study explored the relationship between *CH25H* expression and lung tumor metastasis and its potential mechanism by multidimensional bioinformatics analysis and preliminary confirmation by leukocyte experiments.

## Patients and methods

### *CH25H* expression analyses by public database

GEPIA (http://gepia.cancer-pku.cn/), a newly developed interactive web server was used to analyze the RNA sequencing expression data of 9,736 tumors and 8,587 normal samples from The Cancer Genome Atlas (TCGA) and the Genotype-Tissue Expression (GTEx) projects^11^. This study performed a comparative analysis of the mRNA expression level of *CH25H* in tumor and normal tissues.

UALCAN (http://ualcan.path.uab.edu/) is a comprehensive, user-friendly, and interactive web resource that provides easy access to publicly available cancer omics datasets such as TCGA, MET500, and Clinical Proteomic Tumor Analysis Consortium (CPTAC)^12^. The expression data for *CH25H* based on normal and cancer with non-metastatic and metastatic were obtained from the “Expression” submodule of the “TCGA Gene analysis” module. The Student’s t-test was used to generate a p-value, and statistical significance was set at p < 0.05.

### *CH25H* prognostic value analyses

TIMER (https://cistrome.shinyapps.io/timer/) is a comprehensive resource for the systematic analysis of the abundance of six immune infiltrates (B cells, CD4 + T cells, CD8 + T cells, neutrophils, macrophages, and dendritic cells) across diverse cancer types^13^.

Overall survival (OS) data of *CH25H* in LC were analyzed using TIMER. The high- and low-expression groups were cut off by the median expression of *CH25H*. The Kaplan–Meier (K–M) graph was exported with axis units of months, and the log-rank p-value, hazard ratio (HR), and 95% confidence interval curve are shown in each graph.

### Gene-pathway enrichment analysis of *CH25H*

In this study, the analytical module LinkFinder of LinkedOmics^14^ (http://www.linkedomics.org/) provided co-expression gene sets of *CH25H* in patients with LC. Metascape^15^ (https://metascape.org/) was used to generate gene ontology (GO) enrichment and pathway networks of *CH25H*.

### Immune infiltration analysis

Sangerbox Tools (http://www.sangerbox.com/tool) are a free online platform for comprehensive data analysis. We used Sangerbox Tools to estimate the correlation between *CH25H* expression and immune infiltration level in LC patients according to previous literature^16^ and combined it with data from TCGA for prognosis and immunotherapy analysis.

### Ethics approval and consent to participate

This study was conducted according to the principles of the World Medical Association Declaration of Helsinki and approved by the ethics committee of the second Hospital of Jiaxing (IRB approval No. JYEY2017023). All participants provided written informed consent for this study. Other datasets were retrieved from the published literature, so it was confirmed that all written informed consent was obtained.

### Patient selection and evaluation

The patients included in this study had primary LC treated at the Second Hospital of Jiaxing (Zhejiang, China) from June 2019 to August 2020. The cancer was diagnosed by clinical and X-ray findings and confirmed via histology of tumor biopsies. TNM and final staging was performed as per the American Joint Committee on Cancer guidelines (AJCC) 8th edition. The criteria to judge whether the tumor is metastatic is whether the tumor have invaded bone, brain and other distant organ or lymph nodes, also identified as M1 in AJCC staging system. The inclusion criteria included previously employed surgery and/or standardized treatment and histological confirmation of non-small cell lung cancer (NSCLC). The exclusion criteria included multiple organ dysfunction or a history of organ transplantation, small cell lung cancer (SCLC), unwillingness to participate in the study, and cessation of therapy. Data regarding the clinical characteristics were carefully collected.

### Determination of *CH25H* mRNA expression

The blood specimens (10 mL) from all the enrolled patients, including 15 healthy volunteers (non-cancer individuals) were collected and centrifuged at 2500 rpm for 10 min within 2 h of their collection to separate the leukocytes. Total RNA extraction from leukocytes was performed using an RNA Plus Kit (Yunying, Jiaxing, China). Reverse transcription was performed using the HiScript® III 1st Strand cDNA Synthesis Kit (Vazyme, Nanjing, China), and qRT-PCR analysis was performed using the Alldetect™ kit (Yunying, Jiaxing, China) on an ABI 7500 system (ThermoFisher, MA, USA). The TaqMan assay was used for qRT-PCR amplification detection. Ribosomal protein LO (RPLO) was used as the reference gene, and primers and probes (Table S1) were designed using Primer Premier 5.0 (Premier Biosoft, CA, USA), and synthesized by General Biotech (Shanghai, China). Each process was strictly performed according to the manufacturer’s protocol.

### Statistical analyses

The expression level of *CH25H* across different LC groups was analyzed by the Mann–Whitney U test. Statistical analyses were performed with SPSS v.22.0 (IBM Corp., New York, USA). Statistical graphs were generated by the R package “ggplot2”. A P < 0.05 was considered statistically significant and was used as the inclusion standard in the analysis.

## Results

### *CH25H* expression level downregulated in LUAD and correlated with prognosis

**Figure 1:**
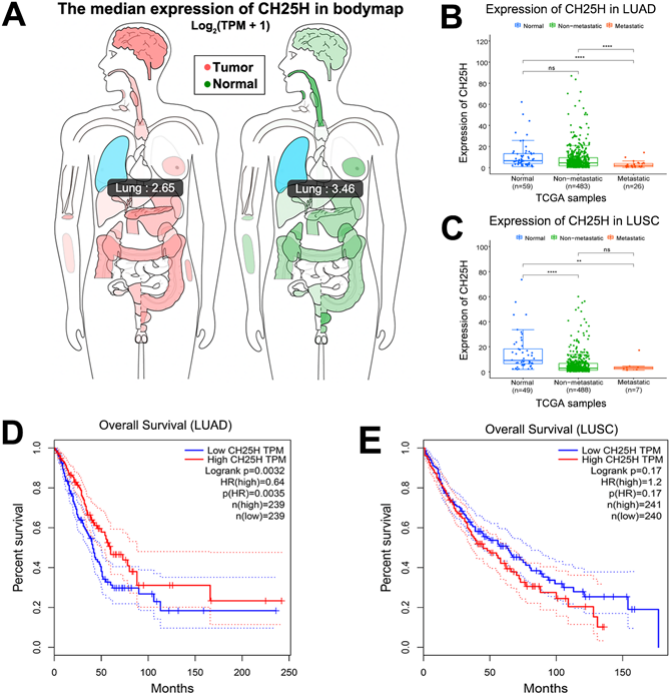
CH25H expression level analysis using public database. (**A**) The bodymap displayed the mRNA expression of *CH25H* in lung tumor and paired normal tissues (GEPIA); (**B**,**C**) the *CH25H* expression levels in normal and tumor with non-metastatic and metastatic tissues (UALCAN). (**D**,**E**) the prognostic value of *CH25H* expression in LUAD and LUSC patients (TIMER). “**”, “***” and “ns” indicate p < 0.01, p < 0.001 and p > 0.05, respectively.

We analyzed the expression level of *CH25H* in lung tumor tissues and normal tissues using online public databases (Fig. 1A). The *CH25H* expression levels showed highly significant differences (p < 0.001), the *CH25H* mRNA expression level in the metastatic group was significantly down-regulated in LUAD compared to the non-metastatic group (Fig. 1B). Compared with normal tissues, *CH25H* expression was significantly down-regulated in the metastatic group (p < 0.001), however, no significant difference was found between the non-metastatic group in LUAD (Fig. 1B). In LUSC, we found that the expression level of *CH25H* was significantly lower than that in normal tissues in both metastatic and non-metastatic groups, but no significant difference was found between the metastatic and non-metastatic groups (Fig. 1C). We observed that in LUAD, this negative expression was significantly associated with sex (Fig. S1B), and nodal metastasis status (Fig. S1F). Other factors such as age (Fig. S1A), race (Fig. S1C), smoking habits (Fig. S1D), and histological subtypes (Fig. S1E) showed poor correlation with *CH25H* expression. However, in patients with LUSC, no outstanding relationship between *CH25H* expression and these factors was observed (Fig. S2).

The survival difference was evaluated, and results showed that compared with the low expression cohort, patients in the high expression cohort showed a significantly enhanced OS (P < 0.01, N = 478), indicating a better prognosis (Fig. 1D). However, compared with the lower expression cohort, LUSC patients with high *CH25H* expression showed a reduced trend of survival time in both OS, although the difference was not statistically significant (P > 0.05, N = 481, Fig. 1E).

### *CH25H* associated with immune regulation

**Figure 2:**
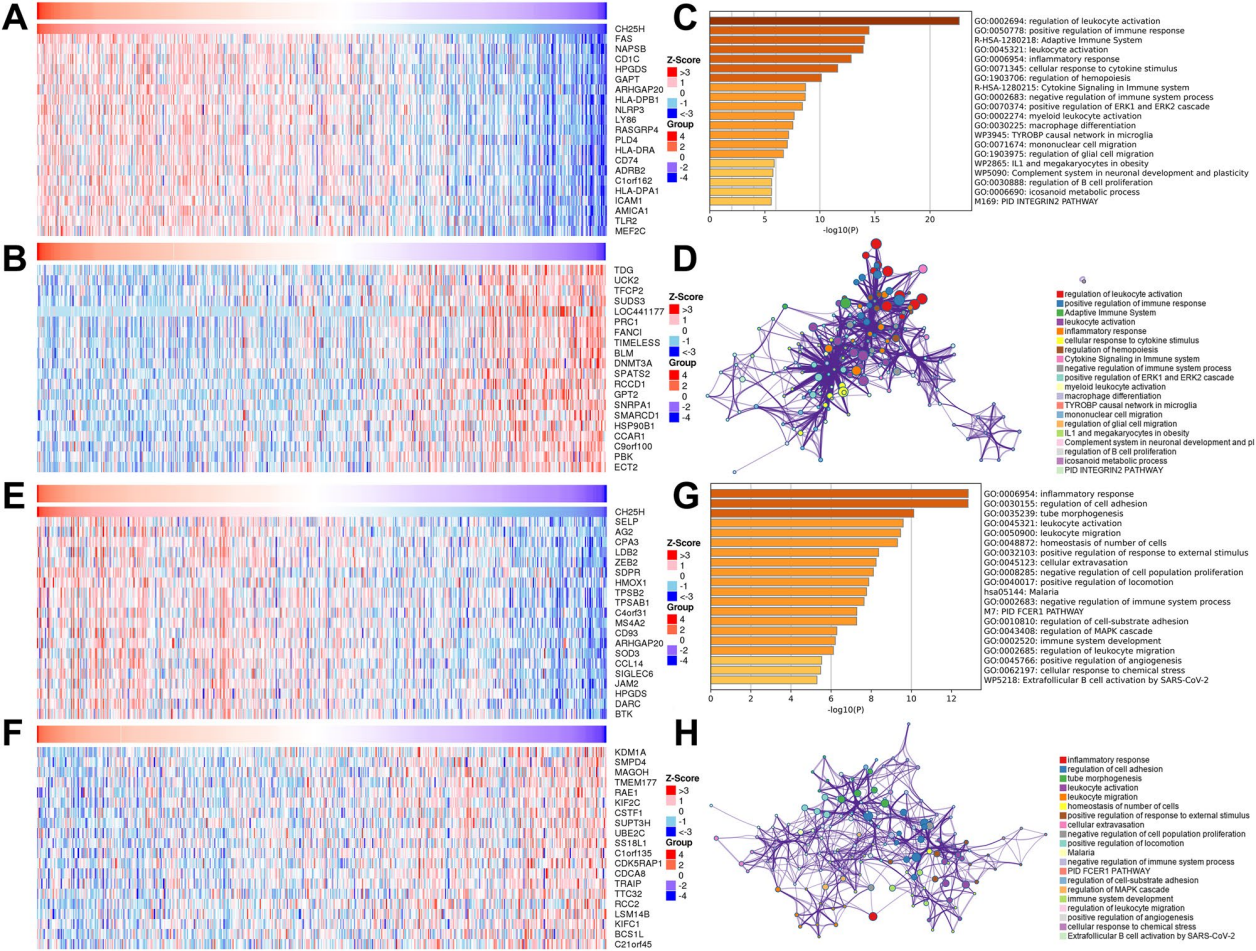
CH25H gene-pathway enrichment in lung cancer. (**A**,**B**,**E**,**F**) heat maps showing the top 20 genes positively and negatively related to *CH25H* in LUAD (**A**,**B**) and LUSC (**E**,**F**) (LinkedOmics). Red shows positively correlated genes and blue demonstrates negatively correlated genes; (**C**,**D**,**G**,**H**) gene ontology (GO) function enrichment of *CH25H* in LUAD (**C**,**D**) and LUSC (**G**,**H**) (Metascape).

Heat maps showed that the top 20 significant genes were positively and negatively related to *CH25H* in LUAD (Fig. 2A,B) and LUSC (Fig. 2E,F), respectively. Notably, 6/20 genes (*CD1C*, *CD74*, *HLA-DPB1*, *NLRP3*, *ADRB2* and *AMICA1*) were positively related to *CH25H* in LUAD. They were therefore responsible for the positive regulation of the immune system^17–22^. Pathway analysis revealed an enriched immune regulation, such as the regulation of leukocyte activation, positive regulation of immune response, and adaptive immune system (Fig. 2C,D). However, in LUSC, *CH25H* showed a weak association with the immune regulation, as analyzed by gene enrichment (Fig. 2E,F) and pathway analysis (Fig. 2G,H). These results could possibly explain the importance of *CH25H* as a prognostic biomarker in case of LUAD only. It was therefore hypothesized that *CH25H* controls the spread and infiltration of tumor cells via immune regulation in LUAD.

Some studies have found that biomarkers such as *CLEC3B*^26^ and *YTHDF1*/*YTHDF2* ^27^ affect the immune microenvironment, and metastasis and prognosis of lung cancer. Therefore, we studied the correlation between *CH25H* expression and immune cell infiltration.

### *CH25H* expression level associated with immune infiltration level

**Figure 3:**
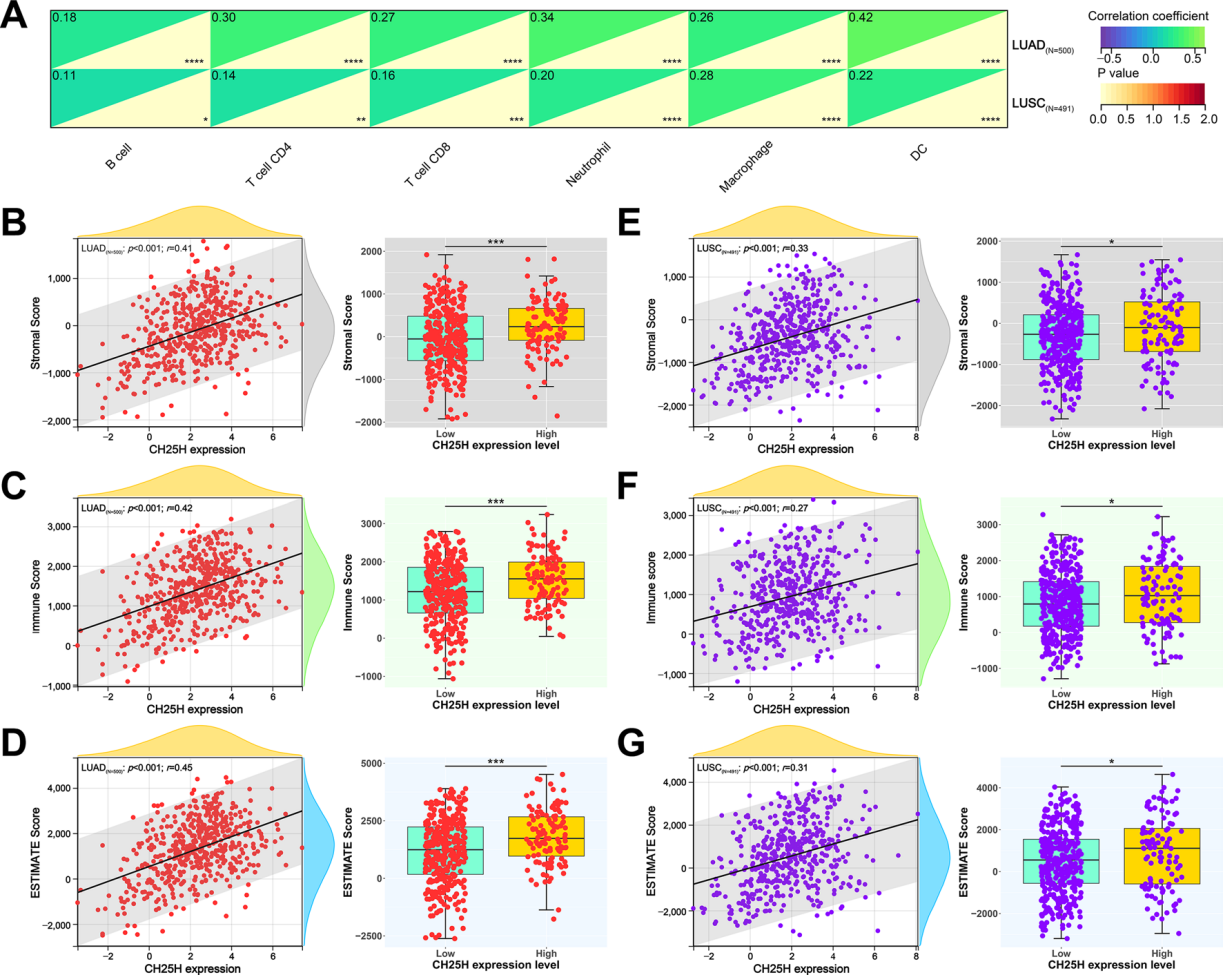
CH25H immune infiltration. (**A**) *CH25H* expression levels had a significant positive association with the infiltration level of B cells, CD4 + T cells, CD8 + T cells, dendritic cells, macrophages and neutrophils in LUAD; (**B**–**G**) the expression levels of *CH25H* had a significant positive relationship with the immune score of LUAD samples. Box plots were generated by R package “ggplot2” using data from Sangerbox. “*”, and “***” indicate p < 0.05, and p < 0.001, respectively.

We hypothesized that *CH25H* expression regulates the infiltration levels of immune cells. We also investigated the relationship between *CH25H* expression and the abundance of immune infiltrates. The results showed that *CH25H* expression level was less associated with the infiltration of immune cells (for instance, B cells, T cells, and macrophages) in the LUSC subtype. However, the expression of *CH25H* was significantly positively correlated with immune cell infiltration in LUAD (Fig. 3A). These results confirmed that *CH25H* expression potentially regulates the immune infiltration in patients with LUAD.

In addition, we observed that *CH25H* expression was significantly correlated with immune infiltration in LUAD (Fig. 3B,D), better than in LUSC (Fig. 3E–G), as evaluated by stromal, immune, and comprehensive ESTIMAT, respectively.

### Immune infiltration level associated with prognosis in LUAD

**Figure 4:**
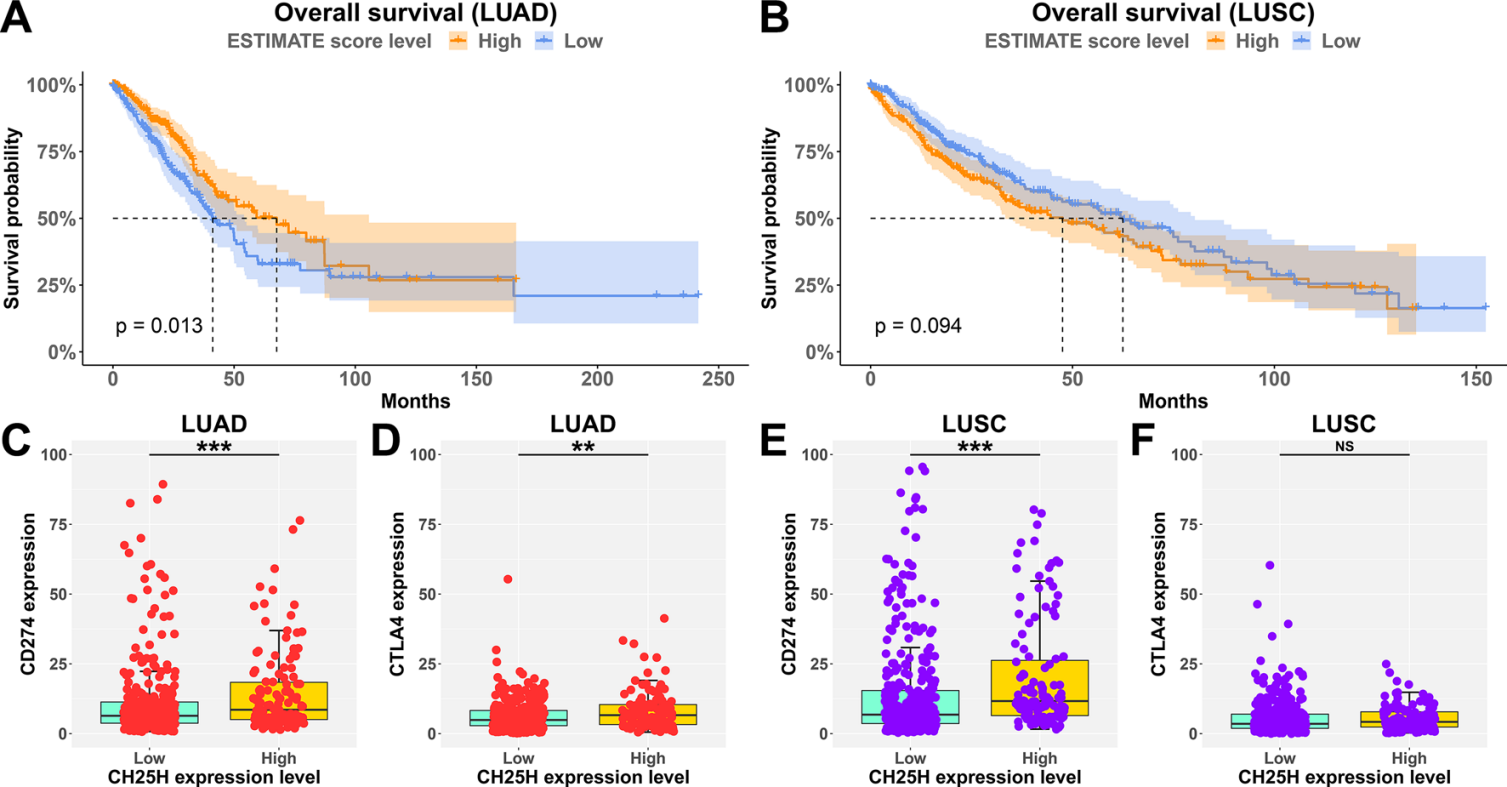
Association of immune score and OS, CH25H expression and immunotherapy markers. (**A**,**B**) Kaplan–Meier survival curves analysis showed that patients with high immune scores had higher overall survival time in TCGA-LUAD; (**C**–**F**) box plot showed that patients with high *CH25H* expression had high immunotherapy markers (CD274 and CTLA4) expression in LUAD. “**”, “***” and “NS” indicate p < 0.01, p < 0.001 and p > 0.05, respectively.

We further investigated the relationship between immune score and OS of LC patients, and the results (Fig. 4A,B) were similar to the result of *CH25H* expressional level and OS (Fig. 1D,E). The survival difference results showed that, compared with the low immune score cohort, the high immune score cohort showed a significantly enhanced OS (P < 0.05) in LUAD (Fig. 4A). Similar analyses were performed for LUSC, but the opposite result was obtained (P > 0.05, Fig. 4B).

### *CH25H* level associated with immunotherapy in LUAD

Immunotherapy molecular marker analysis revealed that both, *CD274* (P < 0.001) and *CTLA4* (P < 0.01) significantly correlated with *CH25H* expression in LUAD (Fig. 4C,D), this was not seen in the case of *PDCD1* (Fig. S3 (left)). However, in LUSC, only *CD274* was significantly related to *CH25H* expression (Fig. 4E) but not *CTLA4* (Fig. 4F) or *PDCD1* (Fig. S3 (right)). The results of this study indicate that *CH25H* can be a potential biomarker for predicting immunotherapy efficacy.

### Leukocytes *CH25H* expression level related to the lung tumor metastasis

To confirm the role of *CH25H* in lung cancer, we conducted a preliminary clinical study using leukocyte samples from patients having lung cancer and non-cancer individuals. This study employed 34 patients newly diagnosed with LC between 2019 and 2020. The average age at the time of diagnosis was 64 years and the median age of the cohort was 65 years (range 38–88 years). Other characteristics of the study participants are listed in Table 1.

**Table 1:** Patient characteristics.

Characteristics	Patients
Total number of patients	34
**Sex**	
Male	23 (67.6%)
Female	11 (32.4%)
**Age at diagnosis (years)**	
≤ 60	9 (26.5%)
> 60	25 (73.5%)
**Primary tumor size (cm)**	
< 2	9 (26.5%)
2–5	21 (61.8%)
> 5	4 (11.8%)
**Primary tumor stage**	
I	4 (11.8%)
II	7 (20.6%)
III	5 (14.7%)
IV	18 (52.9%)
**Histology**	
Adenocarcinoma	23 (67.6%)
Squamous cell carcinoma	11 (32.4%)
**First-line treatment**	
Surgery	11 (32.4%)
Chemotherapy	6 (17.6%)
Surgery + Chemotherapy	17 (50.0%)
**Metastasis**	
Distant Metastasis	18 (52.9%)
Brain metastasis	5 (14.7%)
Bone metastasis	4 (11.8%)
Bone and lymph node metastases	4 (11.8%)
Liver metastasis	5 (14.7%)
Non-metastasis	16 (47.1%)

**Figure 5:**
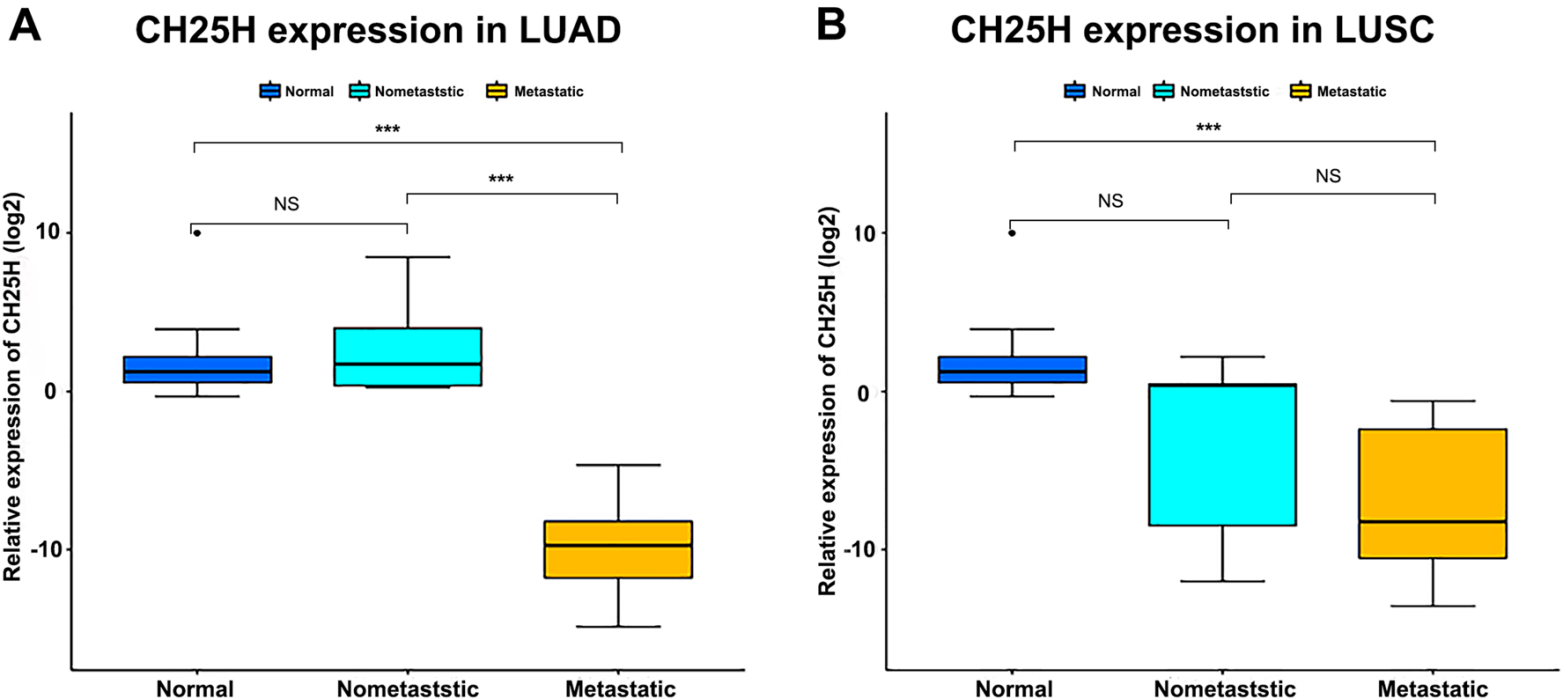
Correlations between lung tumor metastasis and CH25H expression. (**A**) Box plot showed that significant difference (p < 0.001) in *CH25H* expression between metastatic and non-metastatic LUAD patients, and significant difference (p < 0.001) were also found between metastatic group and normal group; (**B**) significant difference were only found between metastatic LUSC patients and normal individuals. “***” and “NS” indicate p < 0.001 and p > 0.05, respectively.

Based on the tumor metastasis status, the patients were divided into metastatic, non-metastatic and normal groups. Blood samples were collected to detect *CH25H* mRNA expression levels in leukocytes. The results showed that, in LUAD patients, *CH25H* expression was significantly decreased in the metastatic group (P < 0.01, Fig. 5A). However, no significant decrease in the CH25H expression was observed in LUSC patients with tumor metastasis (P > 0.05, Fig. 5B), indicating that the *CH25H* expression level is a potential biomarker for LC metastasis prediction, especially in LUAD. Additionally, compared with the normal group, the expression level of *CH25H* in the metastatic group was significantly decreased (P < 0.01), but no significant difference was found in the non-metastatic group, either the LUAD or LUSC dataset.

## Discussion

Previous research has shown that low *CH25H* levels in leukocytes correlate with poor prognosis in melanoma patients^9^. Mechanistically, *CH25H* catalyzes cholesterol oxidation, wherein cholesterol is metabolized to 25-hydroxycholesterol (25-HC) in vivo, which could resist the normal cell absorption of the TEVs (tumor-derived extracellular vesicles), indicating the important role of *CH25H* in defense against the production of the tumor pre-metastatic microenvironment. In this study, we found that low *CH25H* levels were correlated with poor prognosis in patients with LUAD (Fig. 1D).

Genes co-expressed with *CH25H* were analyzed to understand the underlying mechanism of *CH25H* in LUAD. We found that some genes that were positively related to *CH25H* were related to immunity (*CD1C*, *CD74*, *HLA-DPB1*, *NLRP3*, *ADRB2* and *AMICA1*) (Fig. 2A). According to Di Blasio et al., CD1C (+) dendritic cells (DCs) can elicit immunogenic cell death (ICD)^17^. Overexpression of *HLA-DPB2* promotes tumor immune infiltration by regulating *HLA-DPB1*, which is associated with a high survival rate in breast cancer^18^. Lu et al. showed that *NLRP3* inflammasome blockade down-regulates the expression of PD-L1 and reduces the immunosuppression of lymphoma^19^. The expression of *ADRB2* is positively correlated with the infiltration of immune cells in breast cancer, especially the T cells^20^. Activated cGAS-STING signaling in LUAD by *AMICA1* induces immune cell infiltration^21^. High infiltration of CD74 + macrophages in hepatocellular cancer is related to higher infiltration of CD8 + cytotoxic T cells (CTL)^22^. These previous studies suggest that *CH25H* may also be associated with positive regulation of tumor immunity in LUAD. Further, pathway analysis showed that *CH25H* may participate in several immune-related processes, including regulation of leukocyte activation, positive regulation, immune response, and adaptive immune system (Fig. 2C,D), which confirmed our hypothesis.

In our study, according to the Sangerbox tool, we found that *CH25H* is positively related to the immune score and the infiltration of several immune cells, including B cells, CD4 + T cells, CD8 + T cells, dendritic cells, macrophages, and neutrophils (Fig. 3A), which is consistent with the role of *AMICA1* in LUAD according to a previous study^21^. This means that *CH25H* may also affect the level of immune cell infiltration through signaling pathways such as cGAS-STING, however, further research is required. The infiltration level of immune cells is closely related to the effectiveness of immunotherapy and classification basis of “hot” (highly infiltrated) and “cold” (non- infiltrated) tumors^23^. High *CH25H* expression may cause high infiltration level, i.e. “hot” tumor. Furthermore, they may influence the immunotherapy, manifested by an upregulation of the level of immunotherapy molecular markers (such as *CD274* and *CTLA4*, Fig. 4, left plot) in LUAD. Moreover, CD8 + T cells, one of the most important effector cells, play important roles in clearing intracellular pathogens and tumors^23^. So, mechanistically, these results indicated that *CH25H* may affect tumor progression via immune regulation in LUAD. This may also explain the reason behind the decreased *CH25H* expression levels with the progression of LUAD (Fig. 1B).

Moreover, leukocyte *CH25H* expression analysis revealed that *CH25H* expression levels were significantly down-regulated in the metastatic cohort. The main product of *CH25H* catalyzed reaction is 25-HC, which has been reported to suppress the migration and proliferation of gastric cancer cells^24,25^, and the opposite effect was observed in LUAD^26^. Our results are consistent with the outcomes of these studies; when the donor suffered from LUAD, decreased *CH25H* expression reduced the 25-HC level, thereby possibly promoting tumor metastasis, as shown in Fig. 5. Our hypothesis may explain this phenomenon. Decreased *CH25H* expression in LUAD may cause low immune cell infiltration, causing tumor cells to escape immune system and promote tumor metastasis, finally leading to poor prognosis, as shown in Figs. 1D and 4A.

However, this study had some limitations. First, further studies consisting of cell experiments and clinical research are required to validate and explore the potential molecular mechanisms underlying the correlation between *CH25H* expression and immune response. In addition, this was a single-center study with a small sample size. Therefore, prospective multicenter studies involving a larger cohort should be undertaken to expand our understanding of the ability of leukocyte *CH25H* expression to distinguish between metastatic and non-metastatic LC patients. Most importantly, tumor tissue experiments are needed to verify the accuracy of leukemia results.

## Conclusion

Here, through bioinformatics analysis, we speculate that *CH25H* expression levels are associated with immune cell infiltration. This was confirmed by the detection of *CH25H* expression in leukocytes. We identified a potential molecular marker for the prognosis and risk stratification of LUAD.

### Clinical practice points

Previous studies have indicated that the expression level of *CH25H* is an independent prognostic factor for predicting distant metastasis of breast cancer and melanoma, however, its prognostic value in lung cancer remains unclear. Our data showed a decreased *CH25H* expression in LUAD. Notably, low expression of *CH25H* in leukocytes is an obvious molecular characteristic of metastatic tumors which results in poor prognosis. In addition, increased *CH25H* expression improves the overall survival time of patients with LUAD, displaying a potential correlation with immune infiltration. Our findings promote an improved understanding of the mechanisms of *CH25H* in lung cancer and demonstrate that leukocyte *CH25H* expression could be used to develop a rapid, inexpensive, and powerful diagnostic strategy for predicting the risk of lung cancer metastasis. This study will also serve as a foundation for future large-cohort studies to verify the significance of *CH25H* expression.

## Supplementary Information

Supplementary Figure S1.

Supplementary Figure S2.

Supplementary Figure S3.

Supplementary Table S1.

## Data Availability

The data that supporting the findings of this study are available on request from the corresponding author. The data are not publicly available due to privacy or ethical restrictions.
