# Supplementary material for: Leukocyte CH25H is a potential diagnostic and prognostic marker for lung adenocarcinoma
Source: Sci Rep. 2022 Dec 23;12:22201. doi: 10.1038/s41598-022-24183-9 (PMC9789102; doi:10.1038/s41598-022-24183-9)
Supplement: Supplementary file 1 — Supplementary Figure S1. [file 41598_2022_24183_MOESM1_ESM.docx]

**
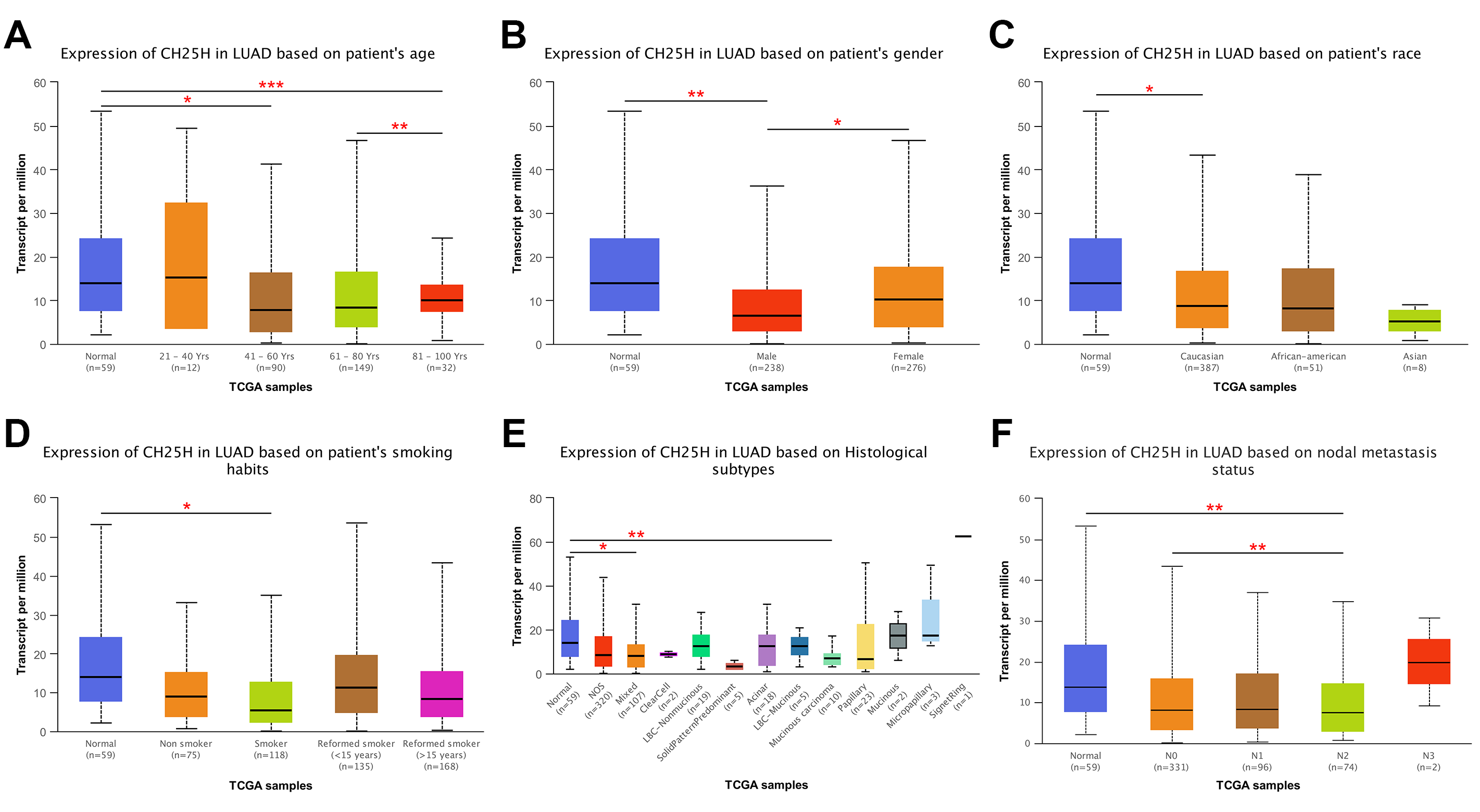
**

**Figure S1.** The transcription of CH25H in LUAD (UALCAN).

The different transcriptional levels of CH25H based on patient’s (A) age, (B) gender, (C) race, (D) smoking habits, (E) histological subtypes (F) nodal metastasis status. “*”, “**”, and “***” indicate p<0.05, p<0.01, and p<0.001, respectively.
