# Supplementary material for: Leukocyte CH25H is a potential diagnostic and prognostic marker for lung adenocarcinoma
Source: Sci Rep. 2022 Dec 23;12:22201. doi: 10.1038/s41598-022-24183-9 (PMC9789102; doi:10.1038/s41598-022-24183-9)
Supplement: Supplementary file 2 — Supplementary Figure S2. [file 41598_2022_24183_MOESM2_ESM.docx]

**
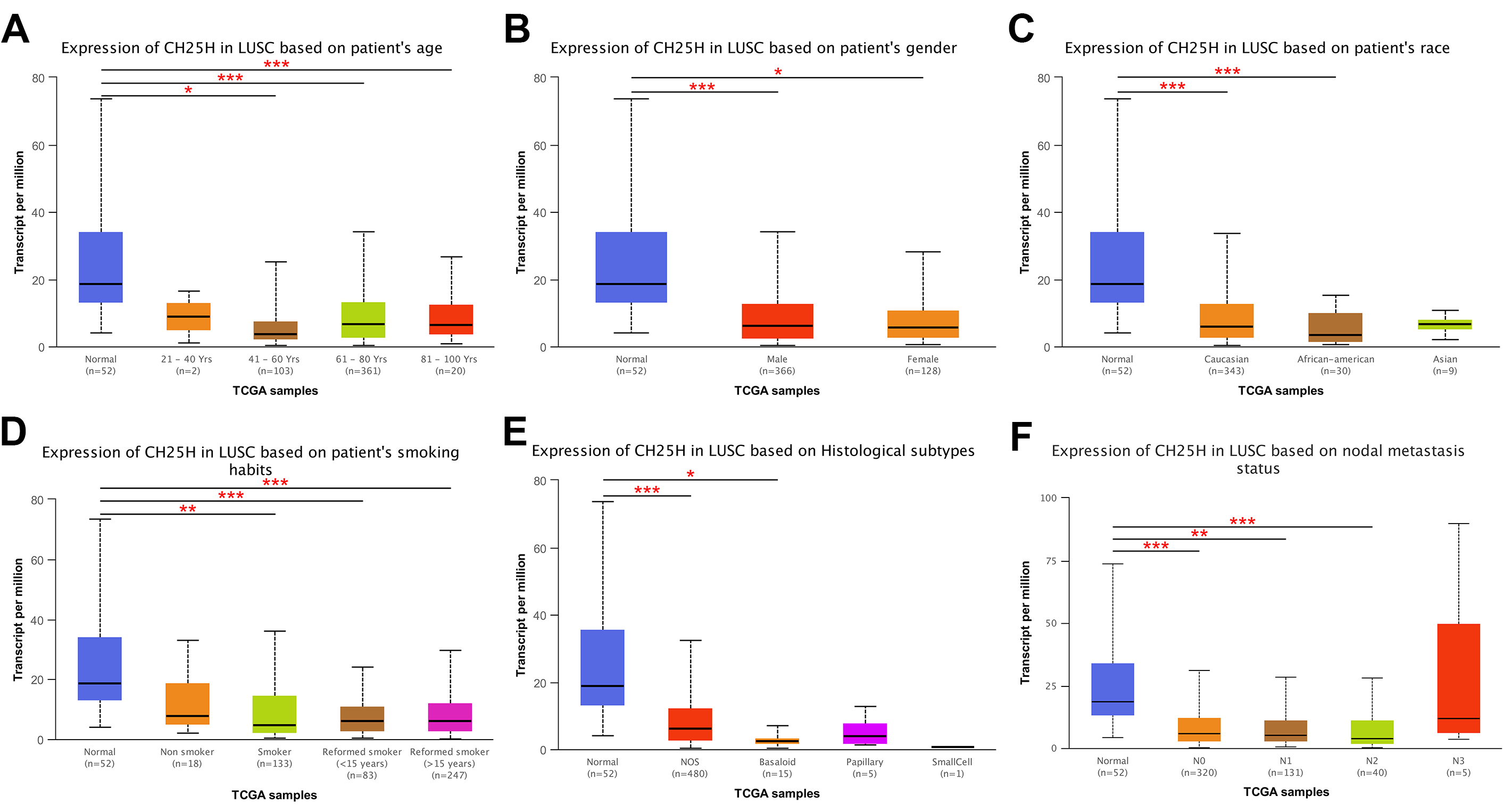
**

**Figure S2.** The transcription of CH25H in LUSC (UALCAN).

The different transcriptional levels of CH25H based on patient’s (A) age, (B) gender, (C) race, (D) smoking habits, (E) histological subtypes (F) nodal metastasis status. “*”, “**”, and “***” indicate p<0.05, p<0.01, and p<0.001, respectively.
