# Supplementary material for: Leukocyte CH25H is a potential diagnostic and prognostic marker for lung adenocarcinoma
Source: Sci Rep. 2022 Dec 23;12:22201. doi: 10.1038/s41598-022-24183-9 (PMC9789102; doi:10.1038/s41598-022-24183-9)
Supplement: Supplementary file 3 — Supplementary Figure S3. [file 41598_2022_24183_MOESM3_ESM.docx]

**
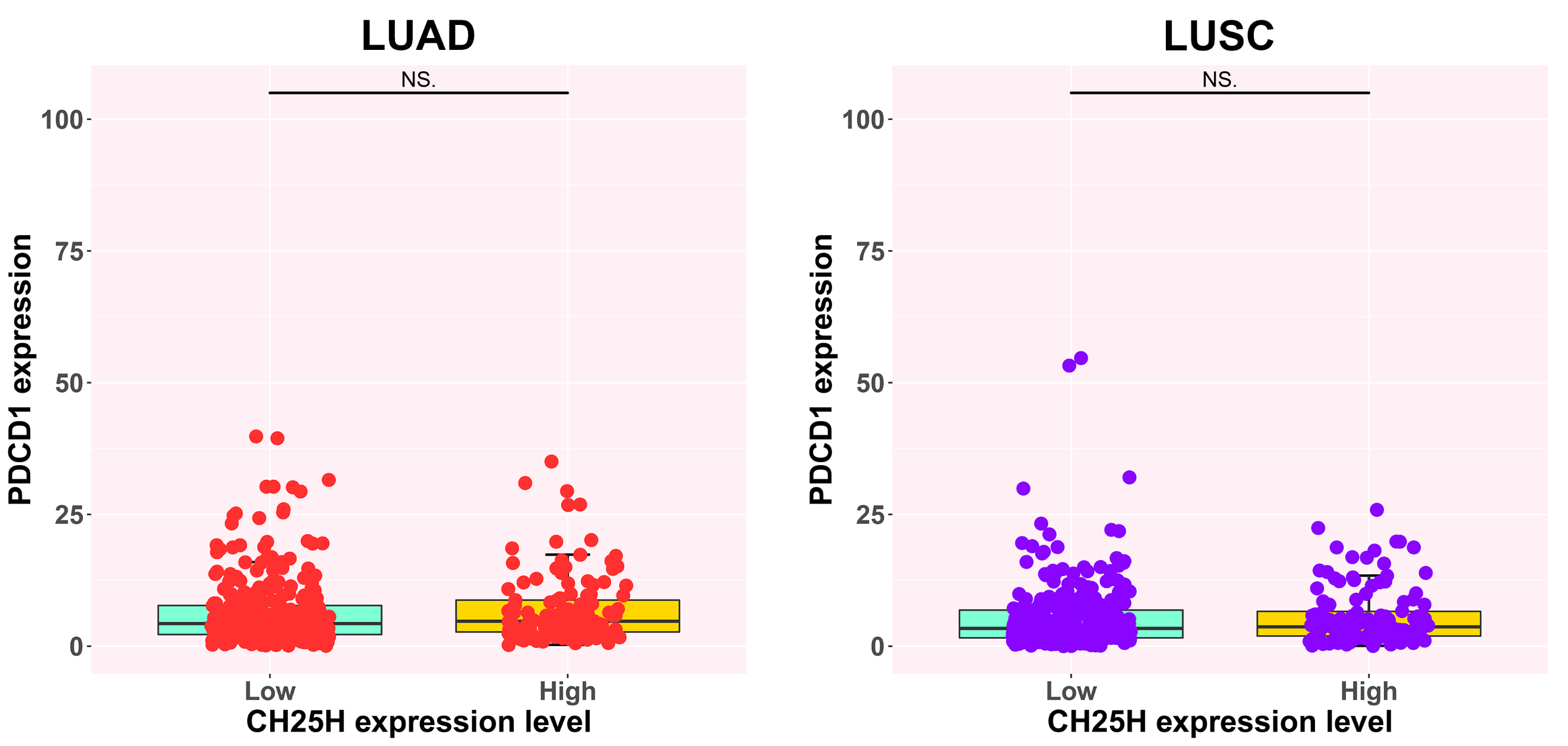
**

**Figure S3. Association of *CH25H* expression and immunotherapy markers**

(A and B) displayed the association of *CH25H* expression and immunotherapy markers in LUAD (left) and LUSC (right) “NS” indicate p > 0.05
