# Supplementary material for: Leukocyte CH25H is a potential diagnostic and prognostic marker for lung adenocarcinoma
Source: Sci Rep. 2022 Dec 23;12:22201. doi: 10.1038/s41598-022-24183-9 (PMC9789102; doi:10.1038/s41598-022-24183-9)
Supplement: Supplementary file 4 — Supplementary Table S1. [file 41598_2022_24183_MOESM4_ESM.docx]

**Table S1. Sequences of primers and probes.**

| Gene | Sequence (5'-3') |
| --- | --- |
| Reverse transcription primer | |
| *CH25H* | CCACATTGTCTGCTCCCACA |
| *RPLO* | ATGTCGAAGAAGCCCAAAGA |
| QPCR primer | |
| *CH25H*-F | AAGGTGCACCACCAGAACTC |
| *CH25H*-R | ATGTCGAAGAAGCCCAAAGA |
| *RPLO*-F | GCGACCTGGAAGTCCAACTA |
| *RPLO*-R | CCACATTGTCTGCTCCCACA |
| Taqman-MGB probe | |
| *CH25H* | CAACGCAGTATATGAGC |
| *RPLO* | CTTAAGATCATCCAACTATTG |

**Notes:**

*Abbr.* F, Forward primer; R, Reverse primer; MGB, minor groove binder.
